# Sociobehavioural Factors Associated With Child Oral Health During COVID-19

**DOI:** 10.1016/j.identj.2022.12.003

**Published:** 2022-12-12

**Authors:** Ravi Kumar Gudipaneni, Mohammed Farhan O. Alruwaili, Kiran Kumar Ganji, Mohmed Isaqali Karobari, Sachin Kulkarni, Kiran Kumar Metta, Ali A. Assiry, Nicholas Israelsson, Omar A Bawazir

**Affiliations:** a Department of Preventive Dentistry, Pediatric Dentistry Division, College of Dentistry, Jouf University, Sakaka, Al Jouf, Saudi Arabia; b College of Dentistry, Jouf University, Al Jouf, Sakaka, Saudi Arabia; c Department of Preventive Dentistry, Periodontics Division, College of Dentistry, Jouf University, Al Jouf, Sakaka, Saudi Arabia; d Center for Transdisciplinary Research (CFTR), Saveetha Dental College & Hospitals, Saveetha Institute of Medical and Technical Sciences University, Chennai, Tamil Nadu, India; e School of Dentistry and Oral Health, University of Adelaide, Adelaide, South Australia, Australia; f Griffith University, Gold Coast, Queensland, Australia; g Department of Conservative Dental Sciences, Ibn Sina National College For Medical Studies, Jeddah, Saudi Arabia; h Preventive Dental Science Department, Faculty of Dentistry, Najran University, Najran, Saudi Arabia; i SA Dental Service, Adelaide, South Australia, Australia; j Department of Pediatric Dentistry and Orthodontics, College of Dentistry, King Saud University, Riyadh, Saudi Arabia

**Keywords:** COVID-19 pandemic, Self-reported child oral health, Self-perceived need of dental care, Dental visiting pattern, Oral health care affordability

## Abstract

**Objectives:**

The aim of this study was to identify the sociobehavioural factors that influenced children's oral health during the COVID-19 pandemic.

**Methods:**

The online cross-sectional study was conducted in Al Jouf Province in the northern region of Saudi Arabia. A total of 960 parents of children aged 5 to 14 years were invited by multistage stratified random sampling. Descriptive, multinomial, and multiple logistic regression analyses were performed to estimate odds ratios and determine the relationship between independent and dependent variables. *P* < .05 was considered statistically significant.

**Results:**

Of the 960 participants, 693 (72.1%) reported that their child had 1 or more untreated dental decay. The children of uneducated parents were 1.6-fold more likely to have 1 or more untreated dental decay (adjusted odds ratio [AOR], 1.66; 95% CI, 0.74–3.73; *P* < .001). The children of unemployed parents were 4.3-fold more likely to have a financial burden for a child dental visit (AOR, 4.34; 95% CI, 2.73–6.89; *P* < .001). Parents from a rural area were 26.3-fold more likely to have spent a lag period of over 2 years since their child's last dental visit (AOR, 26.34; 95% CI, 7.48–92.79; *P* < .001). Nursery-level children were 5.4-fold more likely to need immediate care (AOR, 5.38; 95% CI, 3.01–9.60; *P* < .001).

**Conclusions:**

The present study demonstrated a very high prevalence of 1 or more untreated dental decay in our cohort. Children of rural areas, uneducated, unemployed, widow/divorced, low- and middle-income parents and nursery school children were linked to poorly predictive outcomes of child oral health during the pandemic.

## Introduction

The COVID-19 outbreak affects health care workers and the general population. Its economic and mental health impact continues to unfold, which has been acknowledged.^1^ In addition, the COVID-19 crisis has heightened fear and ambiguity, placing a burden on the oral health care system and resources.^2^ It poses significant concerns to children's oral health, which can affect adults and children differently.^3^^,^^4^ The World Health Organisation Emergency Committee declared a worldwide health emergency on January 30, 2020, and COVID-19 was identified as a pandemic in March 2020, putting the whole world on lockdown and thereby limiting access to dental care in emergencies.^5^ Visiting dental offices has become risky for children and adults, and providing oral health care during the COVID-19 pandemic has placed dental practitioners at a high risk for contracting the virus.^6^^,^^7^

Unprecedented actions to combat the spread of COVID-19 in Saudi Arabia (KSA) have been considered, such as the suspension of communal transportation, closure of public areas, and isolation of patients with confirmed or suspected infection. Furthermore, dental services in KSA were halted from March to August 2020. Consequently, access to dental services for children throughout the nation was temporarily shut down and limited to only emergency dental procedures across the country.^8^ Although dental clinic closure is a necessary precaution, it may have a negative impact on child oral health amongst low-income and disadvantaged communities.^9^

Prior to the pandemic, Saudi children were reported to have poor oral health and a heavy burden of dental caries, with high levels of untreated caries (80%)^10^ and only 18.9% of children attending routine dental visits.^11^ During the pandemic, children did not have access to routine and follow-up dental visits, which had a significant impact on child oral health. Routine, non-urgent follow-up visits are required for child's optimal oral health; they enable early diagnosis and administration of preventative oral health care services.^12^, ^13^, ^14^ Studies have shown that the level of maternal education, income, and preventative mother–child counselling are the primary factors influencing how often should a child attend routine dental checkups.^15^ Another study has reported that child routine dental visits are dependent on their insurance coverage, affordability, child's age, parental attitude, and access to dental treatments.^16^ Although the situation is constantly changing, most of the dental facilities have resumed, providing routine or non-urgent dental care with limited understanding of the impact of COVID-19 on child oral health.

The effects of COVID-19 exposed and aggravated the depth and breadth of social and economic inequality. Hence, the present study was designed to identify the sociobehavioural factors that influence the oral health of children aged 5 to 14, measured with a self-reported questionnaire completed by parents during the COVID-19 outbreak. Self-reported questionnaires can obtain data economically and safely without the risk of virus transmission. Furthermore, this type of questionnaire could be used to collect information for obtaining a quick overview of health care during the pandemic.^17^ However, literature on the sociobehavioural factors influencing child oral health during the pandemic is limited. Thus, evaluating sociobehavioural factors associated with children's oral health is important because such factors can be used in formulating policies for improving child oral health, promoting oral health care, and providing a snapshot view to the regulatory authorities for decision-making and resource allocation during the pandemic. Therefore, this study aimed to determine the sociobehavioural variables that affect child oral health by investigating the self-reported oral health of children (presence of ≥1 untreated dental caries), self-perceived need for care based on parental perspective or experience, pattern of child dental visits, and affordability in accessing dental care amongst a sample obtained from Al Jouf Province in the northern region of KSA.

## Methods

### Study design and setting

The present cross-sectional study was conducted in the province of Al Jouf in the northern part of KSA. The parents/guardians of schoolchildren aged 5 to 14 years were invited to participate in this study on behalf of their children. The Local Committee of Bioethics, Jouf University, KSA, granted ethical permission for this study (04–02–42). The data were collected using Google Forms from January to June 2021. The study was conducted following STROBE guidelines. The data sets generated and/or analysed during the current study are not publicly available due to limitations of ethical approval involving the patient data and anonymity, but they are available from the corresponding author on reasonable request.

#### Conceptual framework

The present study was designed on the basis of Andersen's behavioural model to measure the outcome variables.^18^ The 3 primary components of Andersen's model are predisposing, enabling, and need factors.^19^ Predisposing factors include demographic and social characteristics. Age, gender, race/ethnicity, and social characteristics such as participants’ places of residence and employment, educational attainment, and marital status were considered as demographic and social characteristics in this study. Enabling factors used in this study included financial considerations that affect individuals’ ability to utilise health care services.^19^^,^^20^ Need factors included perceived need for oral health care services amongst children based on parents’ view and experience.^19^^,^^20^

### Sample size calculation

In the present study, sample size calculation was estimated with open Epi software.^17^ Considering that the population size was limited (1,000,000), the expected response rate would be 50%; the margin of error was 5%, and the design effect was 2. The minimum estimated sample size was 768 participants. A total of 960 parents or guardians of schoolchildren were included in the final sample for data analysis to reflect the Al Jouf population.

### Study population and sampling technique

The parents of school children were invited by multistage stratified random sampling method. Parents of school children aged 5 to 14 years were invited to be a part of this study through school social media. Al Jouf Province has 4 districts: Sakaka, Qurayyat, Duma Al Jandal, and Tabarjal. Each district has been divided into 2 sections: urban and rural. In the first stage, the districts were selected as the first strata, and under each stratum (district), one urban and one rural area was chosen randomly using a simple random sampling technique. The list of registered schools from each district's selected urban and rural areas was obtained and numbered sequentially in the second stage. From the list of schools, one school for boys and one school for girls was chosen by a simple random sampling method using a random number generated by a computer. In the third stage, participants were chosen randomly from a list of students from each class. The schools that refused to participate in the study were replaced by another school from the same stratum. The inclusion criterion of this study was Saudi parents/caretakers of school children aged 5 to 14 years. Parents who were not interested in participating in this study and failed to provide written informed consent were excluded from this study.

### Data collection

Data were collected using a self-reported online questionnaire distributed to the parents through school's official social media. The closed questionnaire items used in this study were written initially in English and then translated into Arabic by a native Arabic bilingual translator. The reliability (Cronbach's alpha) of the questionnaire was assessed using 10% of the responses, which was found to be 0.86. Parents/guardians were asked to fill out a closed-ended questionnaire with 3 basic sections. The first section elicited demographic data of participants such as the child gender, nationality, and age and parental education, marital status, occupation, and current residential location. The second section assessed socioeconomic status (SES) of families based on family income: income per month less than 3000 SAR was considered low economic status; income between 3000 and 10,000 SAR was middle economic status, and income more than 10,000 SAR was high economic status.^18^ The third section focussed on oral health behavioural characteristics including self-reported child oral health, self-perceived need for oral care, pattern of dental visits, and oral health care affordability. The questionnaire items of behavioural characteristics are outlined in Table 1.

**Table 1:** Demographic and sociobehavioural characteristics of study participants.

Characteristics	Variables	Participants, No. (%)
Gender of the parent	Male	454 (47.3%)
	Female	506 (52.7%)
Parent age groups	25–30	148 (15.4%)
	30–35	211 (22.0%)
	35–40	227 (23.6%)
	>40	374 (38.9%)
Parental educational level	Uneducated	76 (7.9%)
	Primary school	103 (10.7%)
	Secondary school	232 (24.1%)
	Graduation and above	549 (57.1%)
Parental marital status	Married	836 (87.0%)
	Widowed/divorced/separated	124 (12.9%)
Parental occupation	Unemployed	171 (17.8%)
	Government employee	592 (61.6%)
	Private employee	81 (8.4%)
	Self-employed/own business	116 (12.1%)
Place of residence	Rural	663 (69.0%)
	Urban	297 (30.9%)
Level of child study	Nursery school	580 (60.4%)
	Primary school	380 (39.5%)
Socioeconomic status	Low (≤3000 Saudi riyals)	307 (31.9%)
	Middle (>3000 to <10,000 Saudi riyals)	322 (33.5%)
	High (≥10,000 Saudi riyals)	331 (34.4%)
Self-reported child oral health	Presence of ≥1 untreated decayed teeth	693 (72.1%)
Self-perceived need of oral care	Need of immediate care (dental pain management, extractions, dental trauma)	160 (16.6%)
	Preventive/routine dental services (scaling and polishing, topical fluorides, fissure sealants)	547 (56.9%)
	Advanced treatment options (restorations, root canal treatments, crowns, orthodontic procedure, and others)	253 (26.3%)
Pattern of dental visit	(1) <6 months ago	475 (49.4%)
	(2) 6 months to <1 year ago	165 (17.2%)
	(3) 1 year to <2 years ago	221 (23.0%)
	(4) ≥2 years ago	99 (10.3%)
Oral health care affordability	Dental visit for the last 6 months was a financial burden	377 (39.2%)

#### Statistical analysis

Statistical analyses performed included descriptive statistics, binary logistic regression, and multinomial logistic regression. The binary logistic regression and multinomial logistic regression analyses were performed to determine the variables that are associated with the dependent variables at 2 and 3 levels, respectively. Regression analyses presented the crude odds ratio (COR), adjusted odds ratio (AOR), and their 95% confidence interval (CI).

In the binary logistic regression analysis, all variables with a *P* value <.25 were considered significant and included in the multiple logistic regression analysis. The forwards and backwards selection methods were used in multiple logistic regression to retain the final variables with *P* values <.05. Furthermore, the fitness of the final model was evaluated using the Hosmer–Lemeshow test and receiver operating characteristic curve.

The multinomial logistic regression analysis (the full model, including all the independent variables) was run, and the variables with higher *P* values were removed manually until a parsimonious model was obtained. All data analyses were performed with SPSS version 24 (IBM Corp.). *P* < .05 was considered statistically significant.

## Results

Demographic and sociobehavioural details of the study participants are shown in Table 1. In the current study, 506 (52.7%) of the participants were mothers. The majority of respondents (n = 663, 69.0%) were from rural areas. Of the 960 participants, 693 (72.1%) parents reported that their children have 1 or more untreated dental decay, and 377 (39.3%) respondents reported that dental visits in the last 6 months were a financial burden for the family.

### Self-reported child oral health

Table 2 illustrates the multiple logistic regression model in determining the variables linked to self-reported child oral health with 1 or more untreated dental decay. Children of uneducated parents were 1.6-fold more likely to have 1 or more untreated dental caries (AOR, 1.66; 95% CI, 0.74–3.73; *P* < .001). The children of unemployed parents were 2.1-fold more likely to have 1 or more untreated dental caries (AOR, 2.11; 95% CI, 1.24–3.58; *P* = .006). However, children of parents with middle SES were 3.1-fold more likely to have 1 or more untreated dental caries (AOR, 3.18; 95% CI, 1.96–5.17; *P* < .001). Nursery school children were 2.3-fold more likely to have 1 or more untreated dental caries (AOR, 2.27; 95% CI, 1.55–3.35; *P* < .001).

**Table 2:** Multiple logistic regression model of 1 or more self-reported untreated dental caries.

Characteristics		COR (95% CI)	*P* value	AOR (95% CI)	*P* value
Gender of the parent	Male	0.54 (1.09–0.82)	.538	–	–
	Female (ref.)	1	–	–	–
Parent age groups	25–30 (ref.)	1	–	1	–
	30–35	0.92 (0.58–1.45)	.713	0.72 (0.41–1.24)	.234
	35–40	1.43 (0.88–2.31)	.146	1.26 (0.69–2.29)	.449
	>40	1.02 (0.67–1.56)	.919	1.87 (1.12–3.12)	.017
Parental education level	Graduation and above (ref.)	1	–	1	–
	High school	1.04 (0.73–1.47)	.848	0.65 (0.41–1.02)	.063
	Primary school	0.44 (0.29–0.68)	<.001	0.28 (0.15–0.51)	<.001
	Uneducated	2.18 (1.12–4.25)	.022	1.66 (0.74–3.73)	.218
Marital status	Married (ref.)	1	–	1	–
	Widowed/divorced/separated	0.39 (0.27–0.58)	<.001	0.31 (0.19–0.50)	<.001
Parental occupation	Unemployed	1.45 (0.99–2.13)	.059	2.11 (1.24–3.58)	.006
	Self-employed	2.21 (1.33–3.65)	.002	3.22 (1.75–5.92)	<.001
	Government employee (ref.)	1	–	1	–
	Private employee	3.90 (1.91–7.96)	<.001	3.41 (1.30–9.00)	.013
Level of child study	Nursery school	1.72 (1.29–2.29)	<.001	2.27 (1.55–3.35)	<.001
	Primary school (ref.)	1	–	1	–
Place of residence	Rural	0.61 (0.44–0.84)	.002	0.54 (0.37–0.81)	.003
	Urban (ref.)	1	–	1	–
Socioeconomic status	Low (≤3000 Saudi riyals)	1.09 (0.78–1.52)	.612	1.02 (0.59–1.77)	.935
	Middle (>3000 to <10,000 Saudi riyals)	2.52 (1.74–3.64)	<.001	3.18 (1.96–5.17)	<.001
	High (≥10,000 Saudi riyals) (ref.)	1	–	1	–

AOR, adjusted odds ratio; COR, Crude odds ratio.

### Self-perceived need for child dental care

Table 3 shows the multinomial logistic regression model to evaluate the variables associated with self-perceived need for child dental care during the COVID-19 outbreak. Parents with primary education–level children were 2.6-fold more likely to need immediate oral care (AOR, 2.63; 95% CI, 1.27–5.44; *P* = .009). Children of widowed/divorced parents were 6.2-fold more likely to need immediate dental care (AOR, 6.16; 95% CI, 3.14–12.07; *P* < .001). In addition, children of unemployed parents were 4.9-fold more likely to need advanced care (AOR, 4.91; 95% CI, 3.00–8.06; *P* < .001). Children of self-employed parents were 11.9-fold more likely to need immediate care (AOR, 11.94; 95% CI, 5.65–25.23; *P* < .001). Children of privately employed parents were 21.2-fold more likely to need immediate care (AOR, 21.23; 95% CI, 9.70–46.49; *P* < .001). The parents of nursery-level children were 5.4-fold more likely to need immediate care (AOR, 5.38; 95% CI, 3.01–9.60; *P* < .001).

**Table 3:** Multinomial logistic regression model for self-perceived need for dental care during the COVID-19 outbreak.

Characteristics		Need immediate care AOR (95% CI)	*P* value	Need advance care AOR (95% CI)	*P* value
Gender of the parent	Male	8.39 (4.63–15.19)	.018	1.57 (1.10–2.26)	.014
	Female (ref.)	1	–	1	–
Parent age groups	25–30 (ref.)	1	–	1	–
	30–35	–	–	–	–
	35–40	–	–	–	–
	>40	–	–	–	–
Parental education level	Graduation and above (ref.)	1	–	1	–
	High school	0.72 (0.40–1.28)	.260	0.55 (0.26–1.15)	.111
	Primary school	2.63 (1.27–5.44)	.009	1.05 (0.57–1.93)	.888
	Uneducated	0.31 (0.14–0.72)	.006	0.90 (0.58–1.39)	.623
Marital status	Married (ref.)	1	–	1	–
	Widowed/divorced/separated	6.16 (3.14–12.07)	<.001	1.83 (1.08–3.10)	.024
Parental occupation	Unemployed	5.49 (2.84–10.64)	<.001	4.91 (3.00–8.06)	<.001
	Self-employed	11.94 (5.65–25.23)	<.001	0.90 (0.48–1.70)	.753
	Government employed (ref.)	1	–	1	–
	Private employee	21..23 (9.70–46.49)	<.001	1.03 (0.46–2.27)	.952
Level of child study	Nursery school	5.38 (3.01–9.60)	<.001	0.97 (0.66–1.40)	.850
	Primary school (ref.)	1	–	1	–
Place of residence	Rural	0.29 (0.18–0.47)	<.001	1.10 (0.76–1.59)	.619
	Urban (ref.)	1	–	1	–
Socioeconomic status	Low (≤3000 Saudi riyals)	11.37 (4.93–26.26)	<.001	1.34 (0.78–2.32)	.293
	Middle (>3000 to <10,000 Saudi riyals)	5.44 (2.54–11.63)	<.001	1.11 (0.73–1.70)	.629
	High (≥10,000 Saudi riyals) (ref.)	1	–	1	–

AOR, adjusted odds ratio; COR, Crude odds ratio.

### Pattern of child dental visits

Table 4 illustrates a multinomial logistic regression model to evaluate the variables associated with the pattern of child dental visits during the COVID-19 outbreak. Children of widowed/divorced parents were 6.0-fold more likely to have more than 2 years since their last dental visit (AOR, 5.99; 95% CI, 2.89–12.40; *P* < .001). Children of unemployed parents were 2.2-fold more likely to have a dental visit within 6 months to 1 year (AOR, 2.21; 95% CI, 1.35–3.60; *P* = .002). Moreover, children of self-employed parents were 12.8-fold more likely to have more than 2 years since their last dental visit compared with those who had government-employed parents (AOR, 12.79; 95% CI, 5.55–29.48; *P* < .001). Parents from rural areas were 26.3-fold more likely to have more than 2 years since their child's last dental visit (AOR, 26.34; 95% CI, 7.48–45.79; *P* < .001).

**Table 4:** Multinomial logistic regression model for pattern of dental visit during the COVID 19 outbreak.

Characteristics		6 mo–1 year AOR (95% CI)	*P* value	1–2 years AOR (95% CI)	*P* value	>2 years (AOR 95% CI)	*P* value
Gender of the parent	Male	0.37 (0.24–0.56)	<.001	1.05 (0.72–1.53)	.815	2.35 (1.35–4.11)	.003
	Female (ref.)	1	–	1	–	1	–
Parent age groups	25–30 (ref.)	1	–	1	–	1	–
	30–35	1.61 (0.80–3.24)	.181	0.53 (0.30–0.91)	.023	0.67 (0.31–1.46)	.315
	35–40	1.56 (0.79–3.11)	.201	0.77 (0.45–1.33)	.346	0.61 (0.20–1.81)	.372
	>40	1.18 (0.61–2.28)	.631	0.36 (0.22–0.61)	<.001	1.39 (0.60–3.23)	.439
Parental education level	Graduation and above (ref.)	1	–	–	–	–	–
	High school	–	–	–	–	–	–
	Primary school	–	–	–	–	–	–
	Uneducated	–	–	–	–	–	–
Marital status	Married (ref.)	1		1		1	
	Widowed/divorced/separated	0.87 (0.49–1.55)	.634	1.55 (0.88–2.74)	.129	5.99 (2.89–12.40)	<.001
Parental occupation	Unemployed (ref.)	2.21 (1.35–3.60)	.002	0.29 (0.16–0.51)	<.001	3.06 (1.70–5.52)	<.001
	Self-employed	0.42 (0.22–0.81)	.009	0.04 (0.01–0.14)	<.001	12.79 (5.55–29.48)	<.001
	Government employed	1		1		1	
	Private employee	0.97 (0.52–1.79)	.916	0.14 (0.06–0.33)	<.001	0.80 (0.27–2.40)	.688
Level of child study	Nursery school	0.26 (0.17–0.39)	<.001	0.95 (0.64–1.41)	.803	1.54 (0.78–3.05)	.210
	Primary school (ref.)	1	–	1	–	1	–
Place of residence	Rural	1.42 (0.88–2.29)	.153	0.67 (0.46–0.98)	.041	26.34 (7.48–45.79)	<.001
	Urban (ref.)	1	–	1	–	1	–
Socioeconomic status	Low (≤3000 Saudi riyals)	–	–	–	–	–	–
	Middle (>3000 to <10,000 Saudi riyals)	–	–	–	–	–	–
	High (≥10,000 Saudi riyals) (ref.)	–	–	–	–	–	–

AOR, adjusted odds ratio.

### Oral health care affordability

#### Last 6 months of child dental visits caused a financial burden for families

Table 5 illustrates a multiple logistic regression model in determining the variables associated with oral health care affordability. Children of parents with high school educational background were 1.2-fold more likely to have a financial burden than children of graduate parents (*P* = .408). Children of widowed/divorced parents were 1.7-fold more likely to have financial burden (AOR, 1.68; 95% CI, 1.06–2.66; *P* = .028). Similarly, children of unemployed parents were 4.3-fold more likely to have a financial burden (AOR, 4.34; 95% CI, 2.73–6.89; *P* < .001). Consequently, children of privately employed parents were 15.8-fold more likely to have a financial burden (AOR, 15.81; 95% CI, 7.72–22.37; *P* < .001). Children of parents with low SES were 1.09-fold more likely to have a financial burden (AOR, 1.09; 95% CI, 0.68–1.75; *P* = .715).

**Table 5:** Multiple logistic regression model of dental care affordability during the COVID-19 outbreak.

Characteristics		Financial burden for last 6-month dental visit			
		COR (95% CI)	*P* value	AOR (95% CI)	p-value
Gender of the parent	Male	0.63 (0.48–0.82)	.001	0.67 (0.48–0.94)	.020
	Female (ref.)	1	–	1	–
Parent age groups	25–30 (ref.)	1	–	1	–
	30–35	3.41 (2.13–5.45)	<.001	3.60 (2.03–6.36)	<.001
	35–40	2.30 (1.43–3.72)	.001	1.90 (1.08–3.35)	.026
	>40	2.51 (1.61–3.91)	<.001	3.40 (2.01–5.75)	<.001
Parental educational level	Graduation and above (ref.)	1	–	1	–
	High school	1.30 (0.95–1.77)	.099	1.18 (0.80–1.75)	.408
	Primary school	0.52 (0.32–0.85)	.008	0.50 (0.27–0.92)	.025
	Uneducated	1.81 (1.12–2.93)	.016	0.59 (0.31–1.11)	.099
Marital status	Married (ref.)	1	–	1	–
	Widowed/divorced/separated	1.85 (1.27–2.70)	.001	1.68 (1.06–2.66)	.028
Parental occupation	Unemployed	3.41 (2.40–4.85)	<.001	4.34 (2.73–6.89)	<.001
	Self-employed	1.43 (0.94–2.16)	.094	2.29 (1.40–3.76)	.001
	Government employee (ref.)	1	–	1	–
	Private employee	7.39 (4.33–12.62)	<.001	15.81 (7.72–22.37)	<.001
Level of child study	Nursery school	0.93 (0.72–1.22)	.610	–	–
	Primary school (ref.)	1	–	–	–
Place of residence	Rural	0.92 (0.69–1.21)	.533	–	–
	Urban (ref.)	1	–	–	–
Socioeconomic status	Low (≤3000 Saudi riyals)	2.21 (1.61–3.04)	<.001	1.09 (0.68–1.75)	.715
	Middle (>3000 to <10,000 Saudi riyals)	0.76 (0.55–1.06)	.111	0.56 (0.37–0.84)	.005
	High (≥10,000 Saudi riyals) (ref.)	1	–	–	–

AOR, adjusted odds ratio; COR, Crude odds ratio.

## Discussion

The present study evaluated the sociobehavioural variables linked with child oral health during the COVID-19 outbreak in Al Jouf in the northern region of KSA. Given the feasibility and practical limits of the pandemic, self-reported data were used in this study. In this study, 72.1% of parents reported that their children have 1 or more untreated dental decay. During the pandemic, self-reported dental needs have increased in the Saudi population.^21^ Children of widowed/divorced parents, unemployed parents, and parents with nursery school–level children were, respectively, 6.2-fold, 5.5-fold, and 5.4-fold more likely to need immediate dental care for dental pain management, extractions, and dental trauma in the current study. A study investigated the need for emergency dental treatment in the Saudi population during the COVID-19 outbreak and found that some people were afraid to seek dental treatment.^21^ By contrast, some people were eager to seek care for nonessential and aesthetic concerns.^22^ In many urgent dental situations, most people selected teleconsultations to address dental problems at home instead of going to a dentist's office.^22^ Furthermore, age, gender, socioeconomic factors, accessibility to health care, and perceptions of service quality influenced health-seeking behaviour during the pandemic.^22^

In the current study, privately employed parents were 15.8-fold more likely and unemployed parents were 4.3-fold more likely to have a financial burden for the last child dental visit. This result could be due to the COVID-19 pandemic, thereby affecting the labour market and economy throughout the world. COVID-19 had a significant influence on the Saudi economy in 2020, creating a major spike in the cost of living and worsening living conditions for wide segments of the Saudi populace.^23^ Furthermore, private and public sectors experienced decreased growth rates of 10.1% and 3.5%, respectively, whereas the unemployment rate rose to 15.4% in KSA.^24^ Based on a study from the United States, COVID-19 exacerbates child oral health disparities the most for disadvantaged children.^9^ Unaffordable dental treatment has been demonstrated to have a negative impact on person's health and general well-being.^25^

This study found that parents from rural areas, self-employed parents, and widowed/divorced parents were 26.3-fold, 12.8-fold, and 6.0-fold more likely to have more than 2 years without seeing a dentist for their children, respectively. The oral health care services in KSA were underutilised well before the pandemic.^26^ Based on the American Academy of Pediatric Dentistry, children should visit a dentist regularly, with the frequency of visits primarily determined by the risk of disease or child's individualised needs.^27^ Children in Riyadh, KSA, were reported to have low rates of emergency dental treatment during the outbreak.^28^ The ministry of health in KSA provides free oral health care to the Saudi population. However, in the Saudi population, there is a significant rate of nonregular dental visits that has been related to dental anxiety, a lack of parental motivation, and inadequate oral health literacy.^10^^,^^29^^,^^30^ In addition, parents’ education and awareness play an important role in determining whether to take their children to the dentist for disease-related or preventative treatment.^31^ Therefore, changing this behaviour is critical, and further studies should be planned to assess the barriers in utilising oral health care services in the Saudi population.

Children in the present study have a high prevalence of self-reported dental caries and a high unmet need for dental care. Before the pandemic, the management of children's oral health was a serious concern, although the Saudi population had free access to dental care.^30^ The leading causes of disparities in dental treatment utilisation in North Africa and the Middle East include lack of education, low income, and factors related to health insurance.^32^^,^^33^ In addressing the compromised oral health care delivery system during the current pandemic, oral health care providers and policymakers should integrate teledentistry into routine dental practice.^34^^,^^35^

This study has few limitations. The cross-sectional nature of the study causes difficulty in making causal assumptions, and the data were collected through a self-reported online questionnaire. The self-reported questionnaires are at risk for distortion and bias, such as inaccurate recall and bias due to social desirability.^17^ Moreover, establishing whether financial constraints play a role in poor predicted oral health outcomes is difficult.^36^ Being a child of uneducated, unemployed, widowed/divorced, or low-income parents has been connected to poor oral health outcomes, affordability, dental visit patterns, and self-perceived dental need. Oral health professionals and policymakers should identify the sociobehavioural characteristics linked to children's oral health needs during the COVID-19 pandemic and plan to increase the utilisation of preventive oral health services and promote preventive health-seeking behaviours amongst children and their families to prevent the onset of oral disease and to intervene in a timely manner during a pandemic. Policymakers and oral health care providers should adopt policies to provide primary preventive care and enhance patient education by using technology such as teledentistry. Further longitudinal studies should be conducted to determine the sociobehavioural barriers that affect child oral health in handling future epidemic or pandemic emergencies. The present study recommends that oral health literacy programmes could shift parents’ attitude on accessing dental care and encourage early preventive oral care for children, particularly those at high risk for oral disease and those from low-income families.

## Conclusions

Children in this study had a high prevalence of self-reported dental caries. Children of rural areas, uneducated, unemployed, widowed/divorced and low and middle-income parents and nursery school children were associated with poor predicted oral health outcomes during the pandemic.

## Conflict of interest

None disclosed.

## Appendix Supplementary materials

Image, image 1

Image, image 2
