# Supplementary figures and images for: Sociobehavioural Factors Associated With Child Oral Health During COVID-19
Source: Int Dent J. 2022 Dec 12;73(2):280–7. doi: 10.1016/j.identj.2022.12.003 (PMC9742219; doi:10.1016/j.identj.2022.12.003)

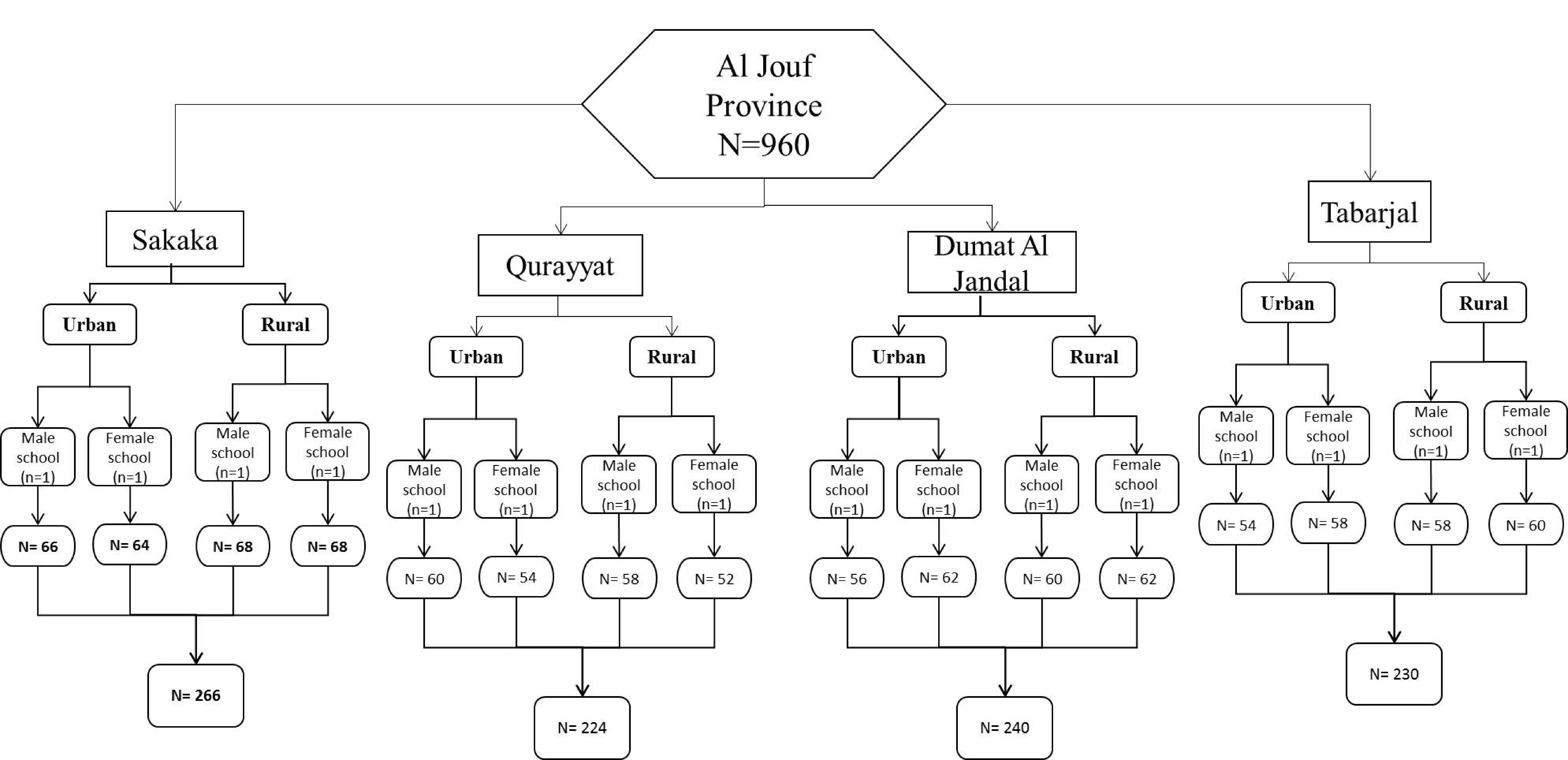

Supplement: Supplementary file 1 [file mmc1.jpg]

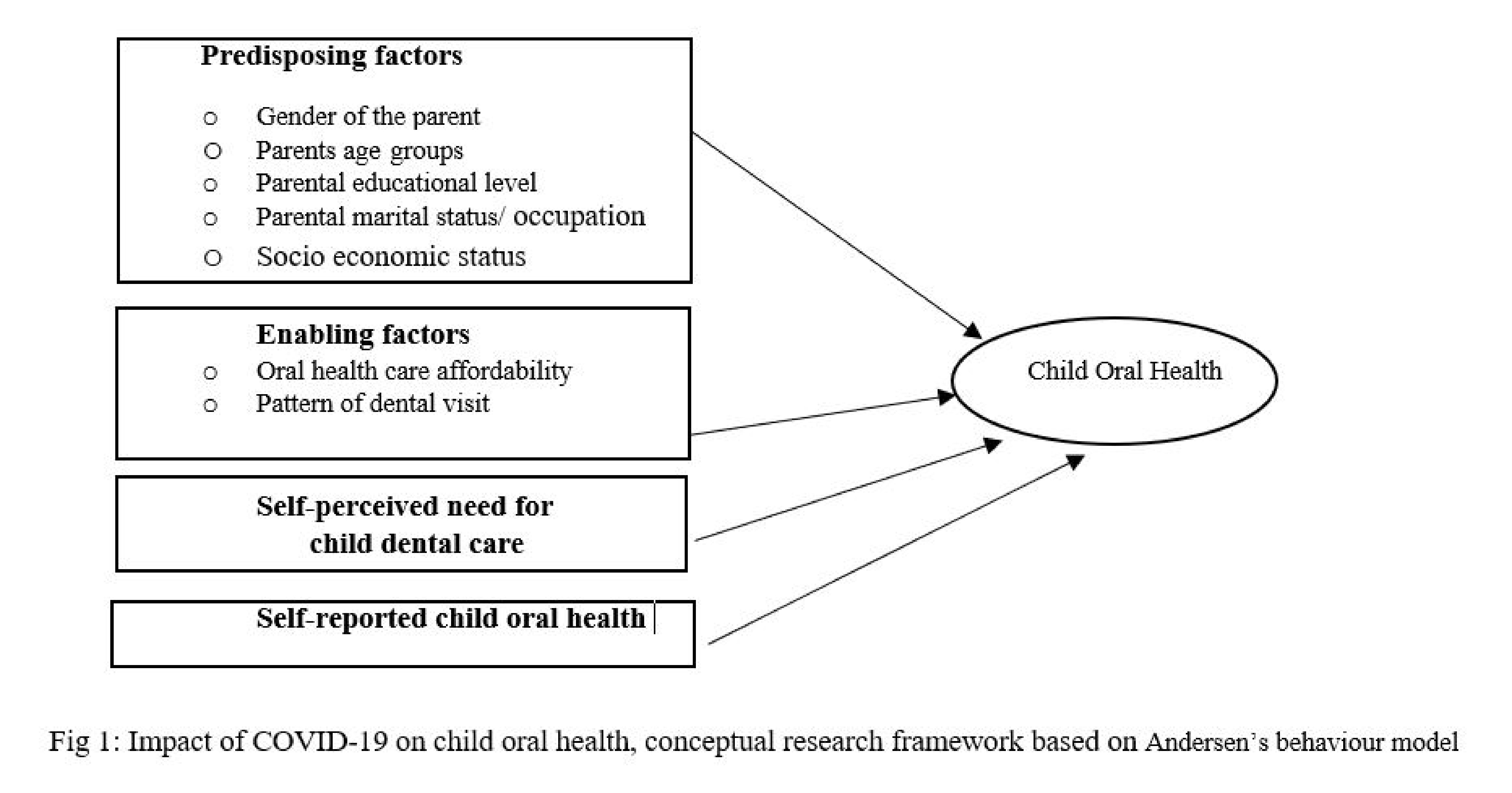

Supplement: Supplementary file 2 [file mmc2.jpg]
